# Schwannoma Located in Nasopharyngeal Region

**DOI:** 10.1155/2016/8260629

**Published:** 2016-05-17

**Authors:** Fadlullah Aksoy, Alper Yenigun, Erol Senturk, Orhan Ozturan

**Affiliations:** Faculty of Medicine, Department of Otorhinolaryngology, Bezmialem Vakif University, Fatih, 34093 Istanbul, Turkey

## Abstract

Schwannoma is a tumor which has neuroectoderm origins, is hard, well-circumscribed, encapsulated, and slow growing benign cranial tumor, and may autonomously grow out of the nerve sheath of peripheral nerves. It is mostly seen in the head and neck region. In the paranasal sinus and nose areas, it is seen at a rate of 4%. The diagnosis is mostly made after histopathological examination. In this paper, a Schwannoma case observed in the nasopharyngeal region was presented in a 20-year-old female who had complaints of sleeping with open mouth, snoring, foreign body feeling in throat, and swallowing difficulties. The tumor was extracted via transoral approach. No recurrence was observed during follow-up over the next year. This case presentation is presented for the first time in the literature in English.

## 1. Introduction

Schwannoma is a tumor which arises from the motor and sensitive peripheral nerve sheath. It consists of Schwann cells and is neuroectodermal in origin, benign encapsulated, and with perineural invasion [1]. Histologically, it is characterized by Antoni A and Antoni B regions and generally occurs in two structures. Furthermore, cellular structures named as Verocay body are available in Antoni A cells. It was first described by Verocay in 1908 [2]. Schwannomas are mostly observed in men and between the ages of 30 and 60 years. They grow slowly in general, cause no pain or symptoms, and are based on the pressure applied by the mass to the peripheral tissues [3].

Approximately 30–40% of Schwannomas are observed in the head and neck region. Most frequently being the Nervous Vagus, they may particularly root in the last fourth cranial nerves and more rarely in autonomous nerves. Schwannomas are also observed in the nasal region at a rate of 4% [4]. Nasal and paranasal sinus Schwannomas originate from the sheath of the ophthalmic and maxillary branch of the trigeminal nerve and from the autonomic nerve system [5]. This case presentation is made since Schwannoma was observed in the nasopharynx for the first time.

## 2. Case Report

**Figure 1:**
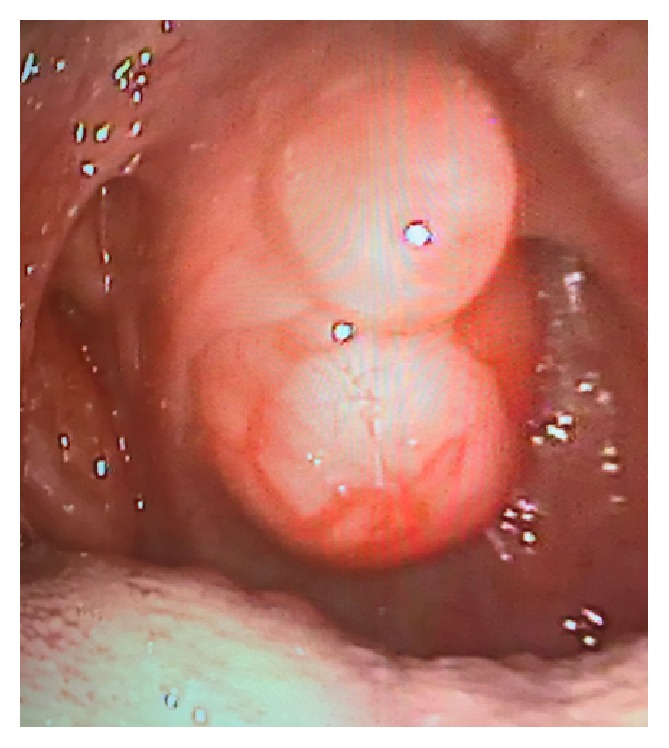
Endoscopic view of the Schwannoma.

A 20-year-old female presented with complaints of sleeping with an open mouth, snoring, a foreign body feeling in the throat, and difficulty in swallowing, which have occurred for 1 year. During her oropharynx and nasal endoscopic examination, a medium hard, semimobile, painless when pressed, and well-circumscribed mass, with 2 × 1 cm dimensions, was observed, originating from the nasopharynx and stretching downwards from the back of the soft palate (Figure 1).

**Figure 2:**
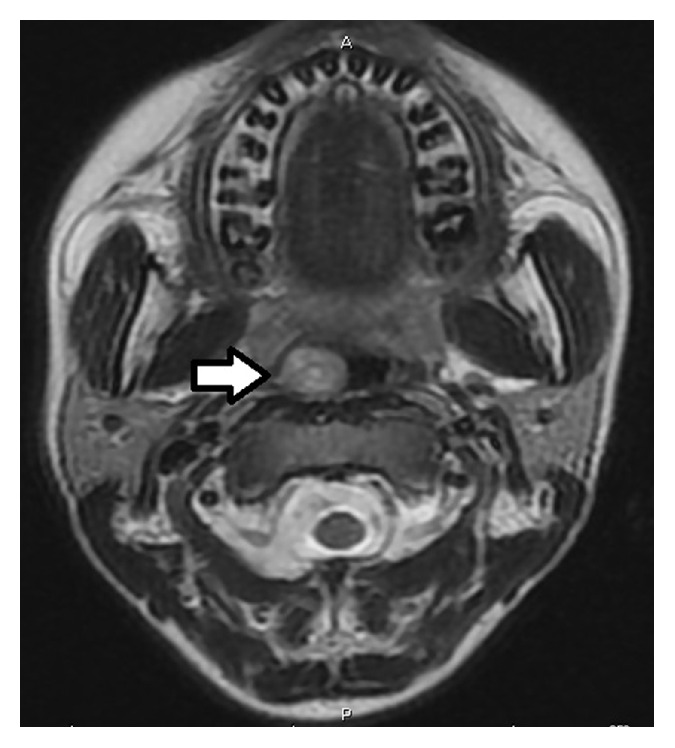
Preoperative axial neck MR.

**Figure 3:**
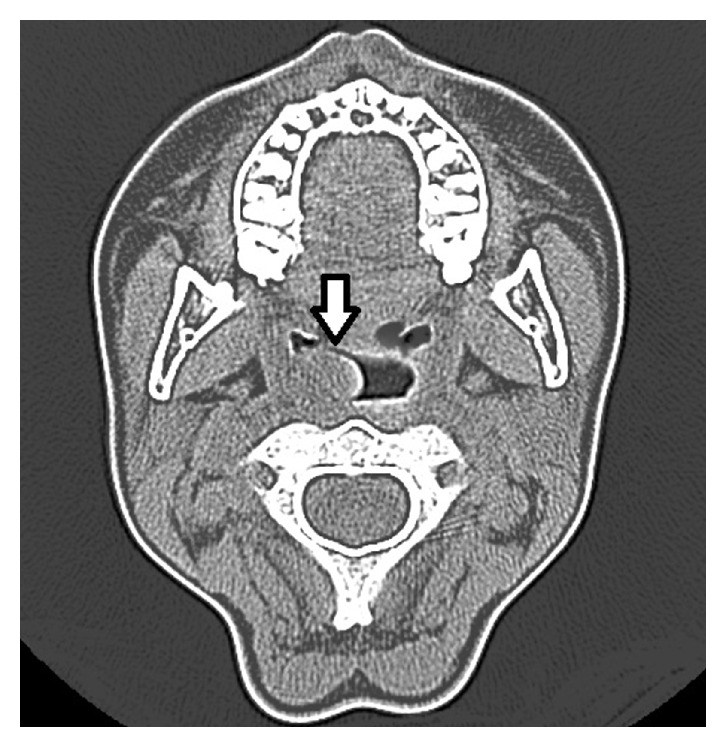
Preoperative axial neck CT.

Magnetic resonance (MR) examination revealed a vascular lesion with no high flow which starts from the nasopharynx and completely fills the choana (Figure 2). In computed tomography (CT) examination, a nonhomogeneous lesion was seen which did not create an erosion in the peripheral tissues and did not reveal calcification (Figure 3).

Apart from these, no pathology was observed in the ear-nose-throat and cranial nerve examinations. The mass was excised transorally under general anesthesia. The mass was well-circumscribed and could be easily distinguished from peripheral tissues.

**Figure 4:**
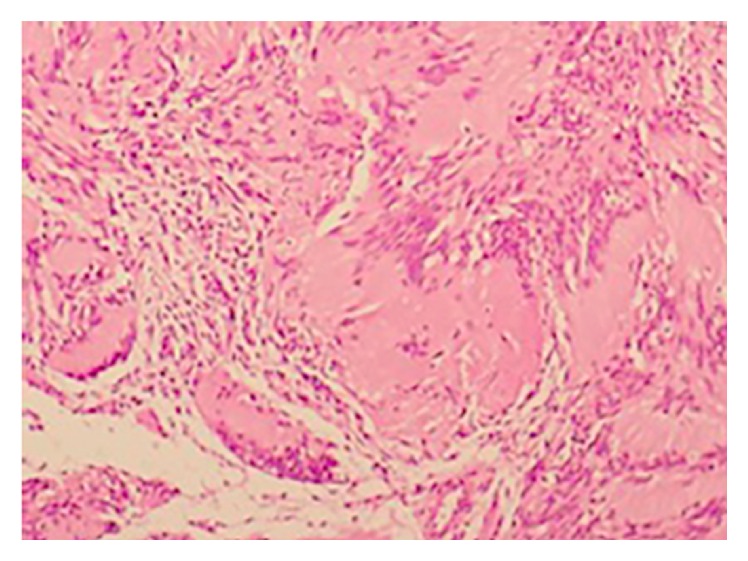
Histopathological view of the Schwannoma.

In histopathological examination, S100 (+) hyalinizing spindle cell mesenchymal tumor, Antoni A, Antoni B, and Verocay corpuscles were identified (Figure 4). The pathological report of the case reported “Schwannoma.” In the postoperative follow-up for one year, no complication or recurrence was observed.

## 3. Discussion

Schwannoma is a benign tumor arising from the nerve sheath or from Schwann cells which do not have a neural element. Although rare, benign Schwannomas might show malign transformation, so it is important to follow up closely [6]. In the differential diagnosis of Schwannomas, parotid tumors, lymphadenopathies, carotid artery lesions, paragangliomas, neurogenic tumors, and other tumors with cranial nerve origin should be considered [7].

In planning diagnosis and treatment, magnetic resonance (MR) is a good choice. In T1 and T2 weighted images, high signal density is remarkable and, unlike paragangliomas, no vascular flow is observed in Schwannomas. Computed tomography (CT) has importance in terms of presenting the lesion's anatomic localization and its relationship with peripheral structures [8]. In MR and CT for our case, heterogeneous density solid mass lesion with proper outline borders was followed up in the nasopharyngeal region.

Since nasal and paranasal sinus Schwannomas grow slowly, they cannot be recognized by nonspecific complaints such as nasal congestion, nasal discharge, nasal bleeding, and swelling of the face [9]. In some advanced cases, they may cause symptoms such as coughing, dysphagia, cranial nerve paralyses, Horner syndrome, and loss of hearing as a result of pressure on peripheral structures [4].

In our case, due to the Schwannoma arising from the nasopharyngeal region, the patient had complaints of sleeping with open mouth, snoring, foreign body feeling in the throat, and swallowing difficulties, which had increased during the last year.

Since the lesion is considered vascular in origin for tumors located around the parapharyngeal region, biopsy is accepted as a relative contraindication [7]. Therefore, no preoperative biopsy was applied in our case.

The gold standard in therapy is that the neural structure of the tumor roots is protected as much as possible and that the tumor is totally excised [10]. In nasal Schwannoma surgery, various techniques are utilized such as lateral rhinotomy, external ethmoidectomy, Caldwell-Luc approach, midfacial degloving, and endoscopic resection [11]. Although gamma or proton radiation is included in alternative therapy options as it stops tumor growth, surgery is a more valuable option since Schwannomas sometimes are resistant to radiation. Prognosis is generally good after surgery. Recurrence may, although rarely, be observed after surgery [12]. In our case, the mass was totally excised via the transoral way and no recurrence was observed during postoperative 1-year follow-up.

In conclusion, Schwannoma should also be considered in the differential prognosis of patients who have sharp and outlined borders with heterogeneous density. In this paper, a Schwannoma case in the nasopharyngeal region is presented for the first time in the literature.
